# Pharmacologic and endotoxic reprogramming of renal vasodilatory, inflammatory, and apoptotic blemishes in weaning preeclamptic rats

**DOI:** 10.1038/s41598-025-87586-4

**Published:** 2025-03-08

**Authors:** Hagar A. Morgaan, Marwa Y. Sallam, Nevine M. El-Deeb, Hanan M. El-Gowelli, Sahar M. El-Gowilly, Mahmoud M. El-Mas

**Affiliations:** 1 Department of Pharmacology and Toxicology, Faculty of Pharmacy, Alexandria University, Alazarita 21521, Alexandria, Egypt; 2 Department of Pathology, Faculty of Medicine, Alexandria University, Alexandria, Egypt; 3 Department of Pharmacology and Toxicology, College of Medicine, Health Sciences Center, Kuwait University, Kuwait City, Kuwait

**Keywords:** Preeclampsia, Endotoxemia, Weaning mothers, Renal vasodilation, Losartan, Pioglitazone, Physiology, Medical research

## Abstract

Preeclampsia (PE) and peripartum sepsis are two complications of pregnancy and are often associated with disturbed renal function due possibly to dysregulated renin angiotensin system. Here we evaluated hemodynamic and renal consequences of separate and combined PE and sepsis insults in weaning mothers and tested whether this interaction is influenced by prenatally-administered losartan (AT1-receptor blocker) or pioglitazone (PPARγ agonist). The PE-rises in blood pressure and proteinuria induced by gestational nitric oxide synthase inhibition (L-NAME, 50 mg/kg/day for 7 days) were attenuated after simultaneous treatment with losartan or pioglitazone. These drugs further improved glomerular and tubular structural defects and impaired vasodilatory responses evoked by adenosinergic (N-ethylcarboxamidoadenosine) or cholinergic (acetylcholine) receptor activation in perfused kidneys of weaning dams. Likewise, treatment of weaning PE dams with a single 4-h dosing of lipopolysaccharides (LPS, 5 mg/kg) weakened renal structural damage, enhanced renal vasodilations and accentuated the upregulated vasodilatory response set off by losartan or pioglitazone. Molecularly, the favorable effect of pharmacologic or endotoxic intervention was coupled with dampened tubular and glomerular expressions of inflammatory (toll-like receptor 4) and apoptotic signals (caspase-3). Our data unveil beneficial and possibly intensified conditioning effect for endotoxemia when combined with losartan or pioglitazone against preeclamptic renovascular dysfunction and inflammation.

## Introduction

Preeclampsia (PE) and sepsis are common causes of pregnancy-related acute kidney injury and share similar pathophysiological features^1,2^. PE is a life-threatening pregnancy-related condition that complicates 2–8% of all pregnancies^3^ and is defined as new-onset hypertension after 20 weeks of gestation together with proteinuria and probably other clinical features of multiple organ damage such as uteroplacental dysfunction, hepatic impairment, renal insufficiency and hematological and neurological complications^4^. Notably, features of renal injury like endothelial dysfunction, proteinuria and glomerular endotheliosis have been demonstrated in some^5^ but not in all^6^ sps:id::bib97 HYPERLINK "sps:refid::bib6" 6reported studies. Evidence also suggests that the mild inflammatory response that naturally accompanies uncomplicated pregnancies is considerably intensified and prolonged following PE due possibly to gigantic upregulation of inflammatory cytokine and chemokine cascades. The latter are blamed for the ensuing placental ischemia and pathophysiologic complications that occur during PE^7,8^. Additionally, the correlated preeclamptic rise in the number of peripheral monocytes and their activity is sought to play a key role in the developed and maintained inflammation^7,9^. Although the normalization of preeclamptic phenotype usually occurs after delivery, PE remains as an imprint predisposing mother to long-term consequences that may reveal far beyond birth^10^.

Like PE, maternal sepsis is another prevalent cause of maternal fatalities worldwide^11^ with more than 75,000 annual maternal deaths^12^. Sepsis is defined as a life-threatening organ dysfunction caused by dysregulated inflammatory response to infection^13^ which can progress to endotoxic shock, a syndrome characterized by hypotension, poor tissue perfusion and multiple system organ failure such as acute renal failure and vascular hyporeactivity^14–16^. Endotoxin or lipopolysaccharides (LPS), a major component of gram-negative bacterial membrane, is one of the experimental models that imitate the inflammatory reaction seen in sepsis^13,17,18^. LPS incites the innate immune system via interaction with toll-like receptor-4 (TLR-4)^19^. PE and sepsis share several similarities in their inflammatory milieus, including strong activation of the innate immune system and the production of pro-inflammatory cytokines like IL-6 and TNF-α. Also, both pathologies involve endothelial dysfunction, oxidative stress, and key immune cells such as monocytes and macrophages^20–23^. Moreover, TLRs play a key role in dysregulating the immune response and inciting inflammation in both endotoxemia^24^ and PE^25^.

The dysregulated renin angiotensin system (RAS) plays a pathogenic role in both PE and endotoxemia. Angiotensin II (Ang II) vasocontriction is reduced during normal pregnancy, but dramatically increases in PE^26^. The upregulation of vascular AT1 receptors elevates peripheral vascular resistance and blood pressure, despite the suppression of the renin–angiotensin–aldosterone system^27,28^. Further, the blockade of angiotensin AT1 receptors diminishes the PE-associated proteinuria, hypertension, and reduced pup weights^29,30^. On the other hand, sepsis is associated with elevated Ang II and depressed AT1 receptor expression, vascular Ang II responsiveness, blood pressure and vascular resistance^31,32^. Moreover, systemic administration of LPS in rodents aberrantly modulates the renal RAS, which may contribute to the deleterious effects of endotoxemia on the kidneys while blood pressure response to exogenous Ang II is markedly reduced^33–36^. Away from its vasoconstrictor properties, RAS activation provokes oxidative stress, endothelial dysfunction, and renal failure^37,38^ where the pharmacologic suppression of RAS is reported to reduce proinflammatory cytokines and improve cardiovascular function and survivability in sepsis^39,40^. To date, current approaches for PE management include antihypertensives, anticonvulsant (magnesium sulfate) to prevent eclamptic seizures, antenatal aspirin in high-risk females and corticosteroids in females (< gestational week 34) to promote fetal lung development^41^. For sepsis, key therapeutic interventions involve administration of antibiotics, restoration of tissue perfusion via fluid resuscitation while concurrently stabilizing the cardiac and respiratory systems^42^.

Despite extensive research on PE and postpartum sepsis as individual entities, little information is available about the renal interaction between these two diseases and possible management. The investigation of these issues is important given the strong relationship between inflammatory milieus of sepsis and PE^43^ and the increased incidence of pathologies like septic pelvic thrombophlebitis in preeclamptic mothers^44^. Therefore, the current study addresses two main questions: (i) What are the individual and joint hemodynamic, renal, and inflammatory consequences of PE and postpartum endotoxemia in weaning mothers? (ii) Are these anomalies favorably impacted by antenatal exposure to the AT1 receptor blocker losartan or the PPARγ agonist pioglitazone? PE was induced by daily administration of the nitric oxide synthase (NOS) inhibitor L-NAME during the last week of gestation^45,46^, endotoxemia was induced by a single i.p. dose of LPS (5 mg/kg) in preeclamptic weaning mothers^18^. Renal and molecular investigations were undertaken to evaluate the impact of antenatal therapies on renovascular derangements elicited by PE alone or combined with endotoxemia. Notably, PPAR agonists are believed to favorably modulate the RAS-mediated control of renal homeostasis^47^ and nullify cardiovascular features of PE^48–50^.

## Results

### Gestational losartan or pioglitazone relieves preeclamptic hypertension and proteinuria

**Fig. 1:**
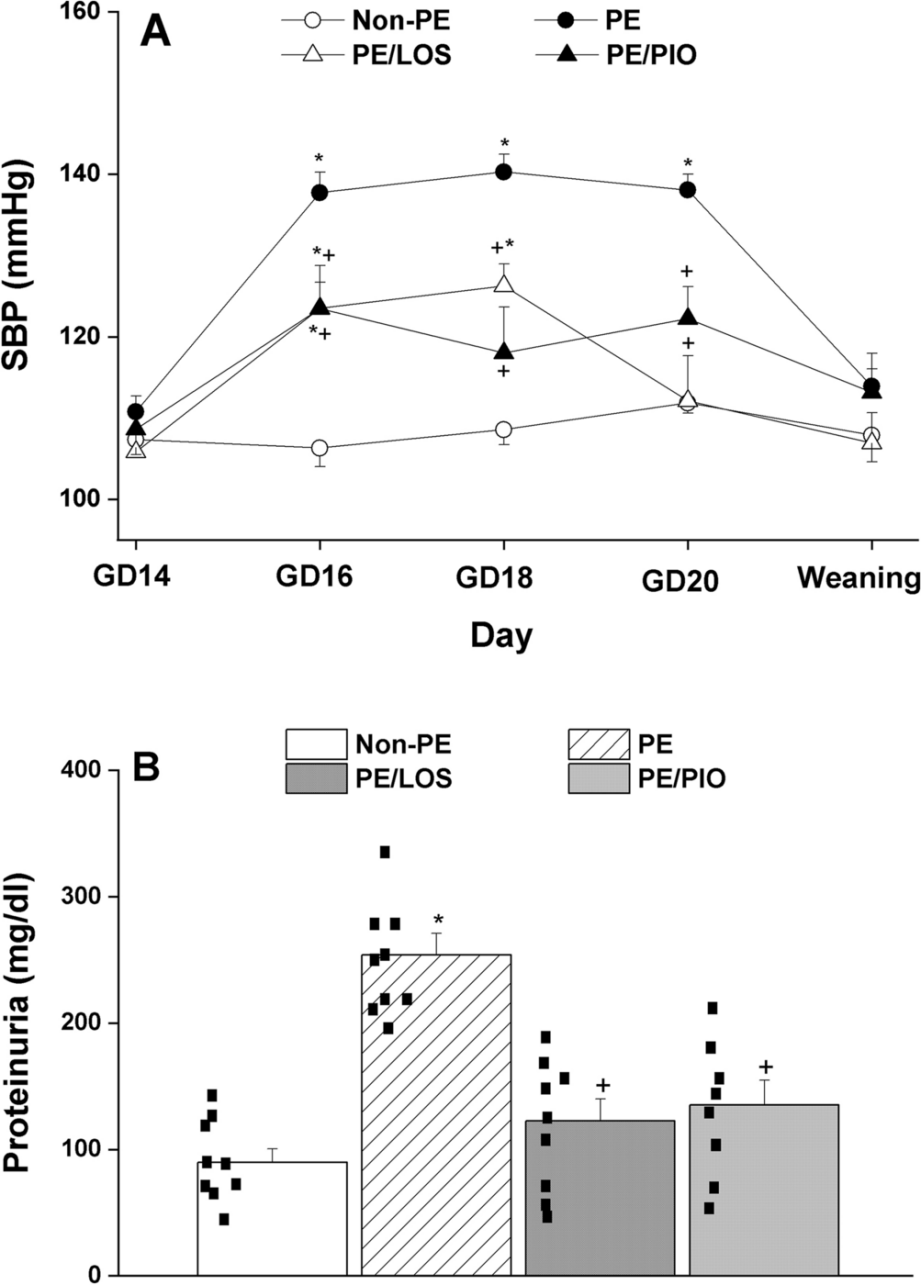
Time course effect of gestational L-NAME administration (50 mg/kg/day, orally) on systolic blood pressure (SBP) in pregnant and 3-week weaning dams measured by tail cuff plethysmography method. The effect of gestational losartan (LOS) (10 mg/kg/day, orally) or pioglitazone (PIO) (5 mg/kg/day, orally) treatments on changes caused by PE insult are also shown (panel A). Urine protein levels measured at gestational day 20 are shown in panel B. Values are expressed as means ± S.E.M of 8–9 measurements. The One-way ANOVA followed by the Tukey’s post hoc was utilized to measure statistical significance. *P < 0.05 vs. “non-PE” and ^+^P < 0.05 vs. “PE”.

The non-invasive tail-cuff plethysmography method was utilized to monitor blood pressure of pregnant and weaning mothers. Figure 1A shows that compared with control (non-PE, n = 8) values, daily injection of pregnant dams with L-NAME (50 mg/kg/day, n = 8) for 7 consecutive days caused significant (p < 0.0001) rises in SBP on gestational day (GD16) and these effects were maintained on GD18 and GD20. The hypertensive response seen in PE rats during gestation disappeared at weaning, i.e. 3 weeks post-labor, where SBP values were not statistically different from those of non-PE dams (Fig. 1A, p = 0.6510). Moreover, tail-cuff measurements demonstrated that gestational treatment of PE rats with losartan (10 mg/kg, n = 8) or pioglitazone (5 mg/kg, n = 8) caused significant (p = 0.0171) reductions in PE-associated elevations in SBP on GD16, GD18, and GD20 (Fig. 1A). At weaning, SBP of PE rats treated prenatally with losartan or pioglitazone was not significantly different from PE (p = 0.5089 and 0.9987, respectively) or non-PE dams (p = 0.9973 and 0.7194, respectively) (Fig. 1A).

The 24-h urine samples collected on GD 20 showed a threefold rise in urine protein levels in PE dams (282 ± 32 mg/dl, n = 9, p < 0.0001) compared with non-PE counterparts (89 ± 10 mg/dl, n = 9). This proteinuric response in PE rats was virtually eliminated following gestational treatment with losartan or pioglitazone (Fig. 1B, n = 8–9, p < 0.0001 and 0.0003, respectively).

### Effects of gestational losartan or pioglitazone on PE/LPS renovascular interaction

**Fig. 2:**
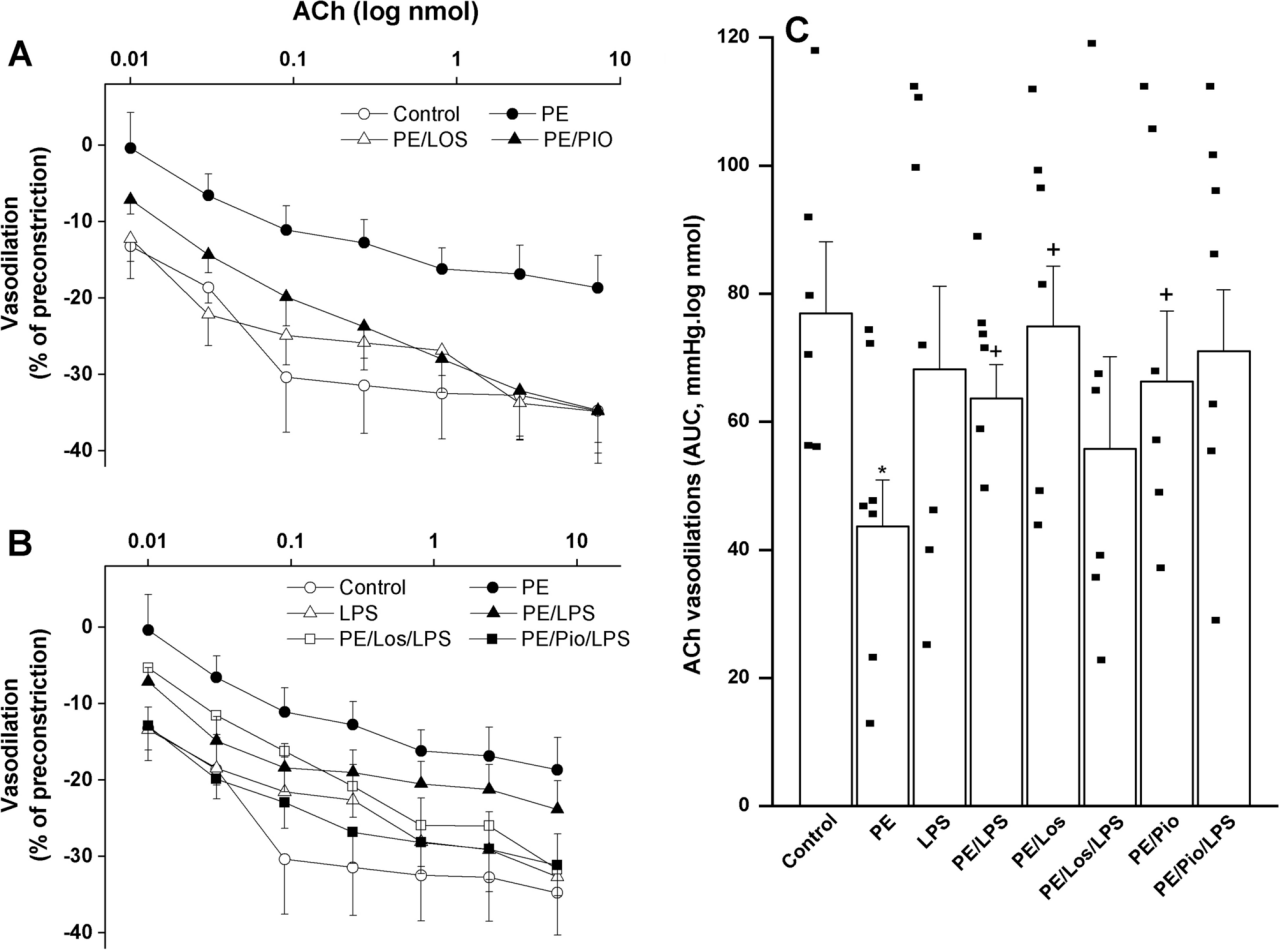
Effect of PE maternal programming on cumulative vasodilatory dose response curves of ACh (panel A & B) and AUCs of the ACh vasodilatory dose response curve (panel C) in phenylephrine preconstricted perfused kidneys of 3 weeks postpartum weaning mothers measured 4 h. post intraperitoneal injection of LPS (5 mg/kg) or equal volume of saline. The effect of gestational losartan (LOS) (10 mg/kg/day, orally) or pioglitazone (PIO) (5 mg/kg/day, orally) treatments on changes caused by PE insult are also shown. Data are expressed as the mean ± SEM of 6–8 measurements. ANOVA followed by the Tukey’s post hoc was utilized to measure statistical significance. *P < 0.05 vs. “Control” and ^+^P < 0.05 vs. “PE”.

**Fig. 3:**
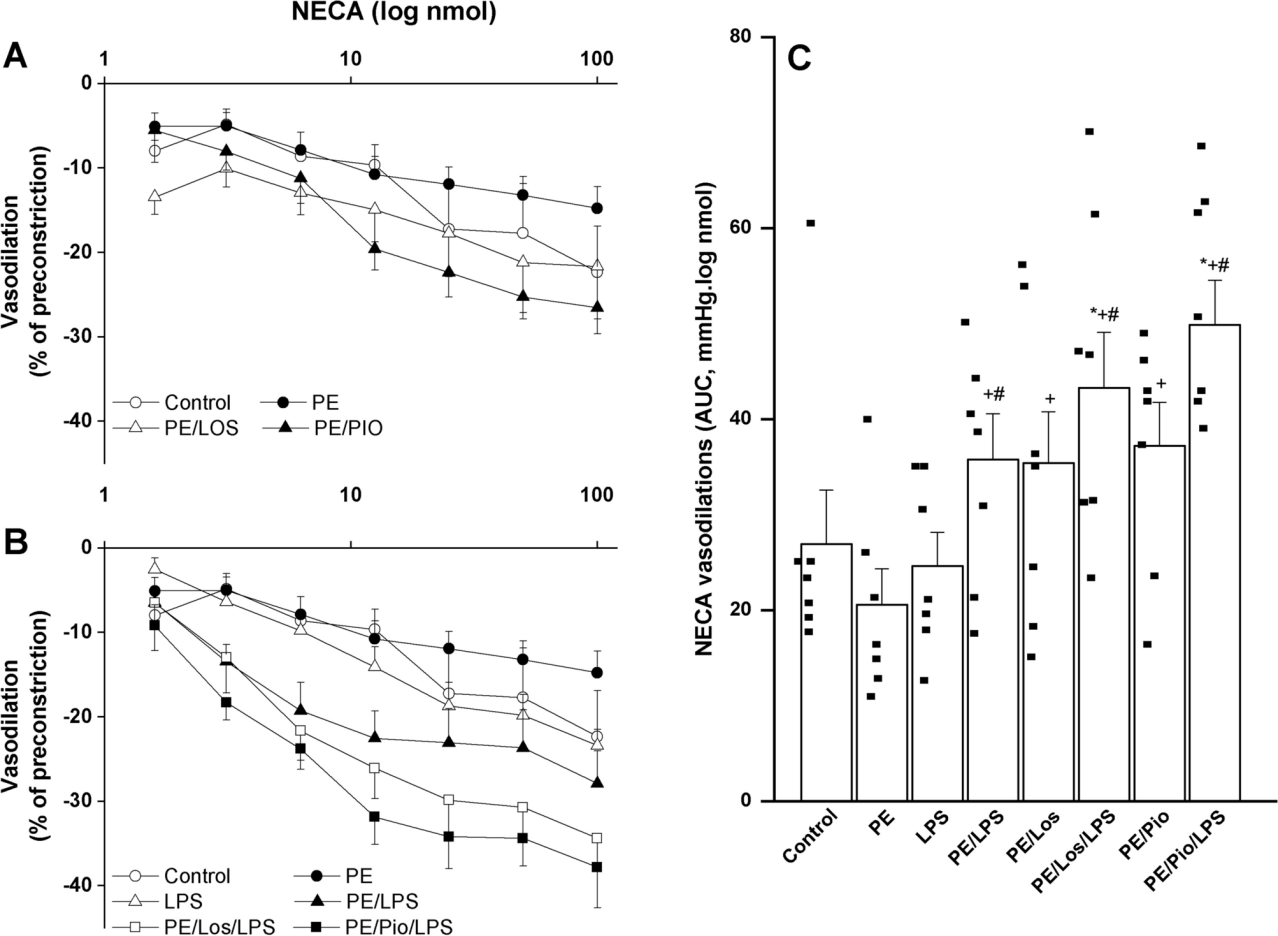
Effect of PE maternal programming on cumulative vasodilatory dose response curves of NECA (panel A & B) and AUCs of the NECA vasodilatory dose response curve (panel C) in phenylephrine preconstricted perfused kidneys of 3 weeks postpartum weaning mothers measured 4 h. post intraperitoneal injection of LPS (5 mg/kg) or equal volume of saline. The effect of gestational losartan (LOS) (10 mg/kg/day, orally) or pioglitazone (PIO) (5 mg/kg/day, orally) treatments on changes caused by PE insult are also shown. Data are expressed as the mean ± SEM of 6–8 measurements. ANOVA followed by the Tukey’s post hoc was utilized to measure statistical significance. *P < 0.05 vs. “Control”, ^+^P < 0.05 vs. “PE” and ^#^P < 0.05 vs. “LPS”.

Isolated perfused kidneys of weaning rats were used to evaluate renal vasodilator responsiveness to cumulative doses of ACh (0.01–7.29 nmol) and NECA (1.6–100 nmol). The aggregate effect of either vasodilator was assessed by calculating AUCs of the vasodilatory dose–response curves. Compared with respective control (non-PE) values (n = 6), perfused kidneys of PE weaning mothers (n = 7) showed upward shifts in dose–response curves of ACh (Fig. 2A-B) that were associated with significant reductions in AUCs of the ACh vasodilatory response (p < 0.05, Fig. 2C). The depressant effects of PE on ACh vasodilations were reversed and near-normal ACh responses were restored after 4-h challenging of weaning PE dams with a single dose of LPS (5 mg/kg, Fig. 2, n = 6), suggesting a postconditioning effect for postpartum endotoxemia. On the other hand, the vasodilator responses elicited by cumulative bolus injections of NECA (1.6–100 nmol) were significantly enhanced in PE/LPS kidneys (n = 7) but remained unaffected by individual interventions (PE or LPS, n = 7 each, p = 0.9852 and 0.9999, respectively) (Fig. 3).

Renovascular studies also showed that antenatal therapies of losartan or pioglitazone significantly increased ACh (Fig. 2, n = 6) and NECA (Fig. 3, n = 7) vasodilations in perfused kidneys obtained from PE dams. Moreover, the treatment with losartan or pioglitazone caused more exaggerated downward shifts in vasodilatory dose–response curves and increases (P < 0.05) in AUCs of NECA (Fig. 3, n = 7), but not ACh (Fig. 2, n = 6–7), in kidney preparations obtained from LPS-treated PE dams.

### Effects of gestational losartan or pioglitazone on renal TLR4/caspase-3 expression and serum NO metabolites

**Fig. 4:**
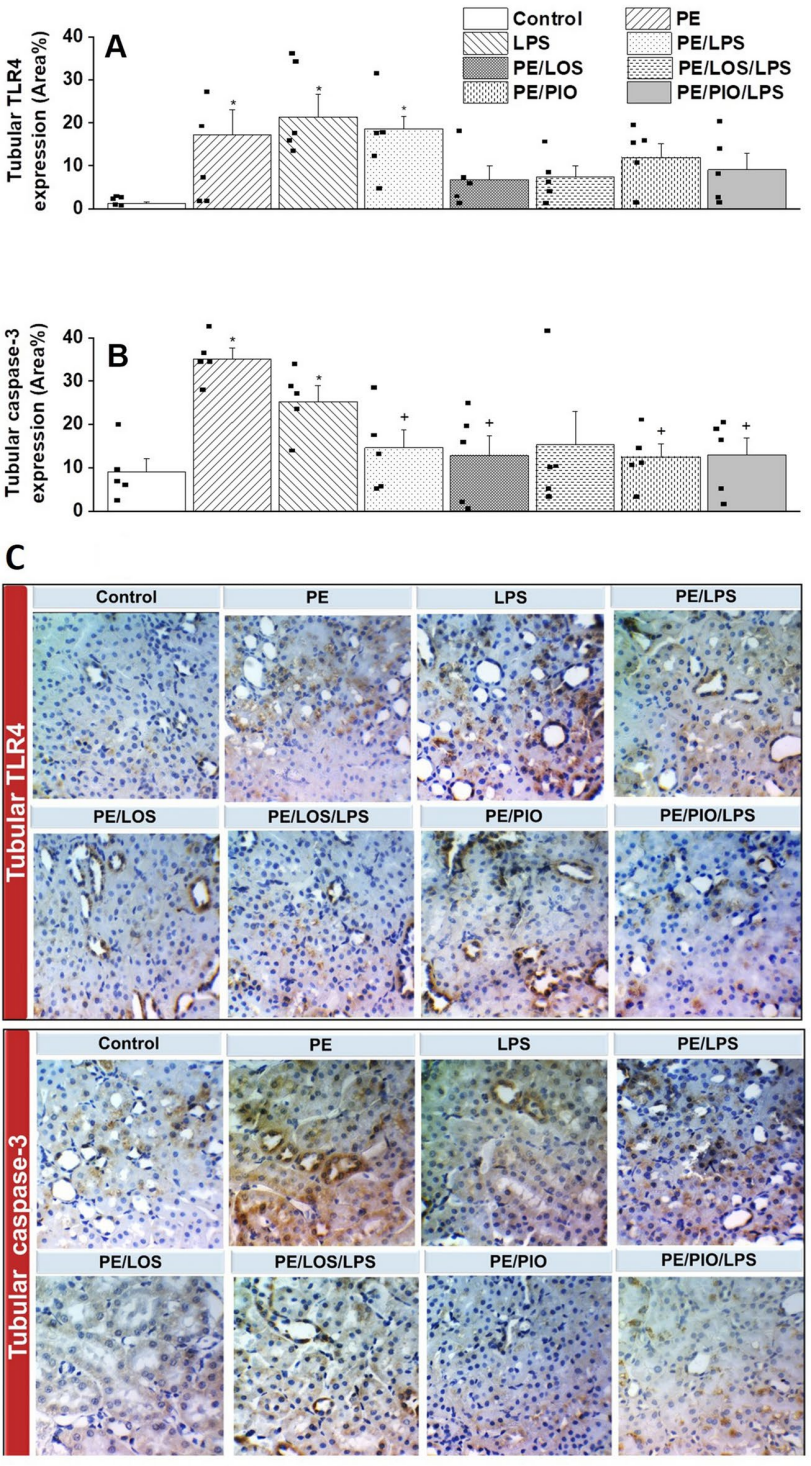
Effect of gestational losartan and pioglitazone therapy on immunohistochemical protein expressions of TLR-4 receptors (panel A) and Caspase-3 (panel B) in renal tubular tissues of PE or PE/LPS dams. Representative images for immunostained renal tubular tissues are shown in panel C. The One-way ANOVA followed by the Tukey’s post hoc was utilized to measure statistical significance. Values are expressed as means ± S.E.M of 5 observations. *p < 0.05 vs. “Control” and ^+^p < 0.05 vs. “PE values.

**Fig. 5:**
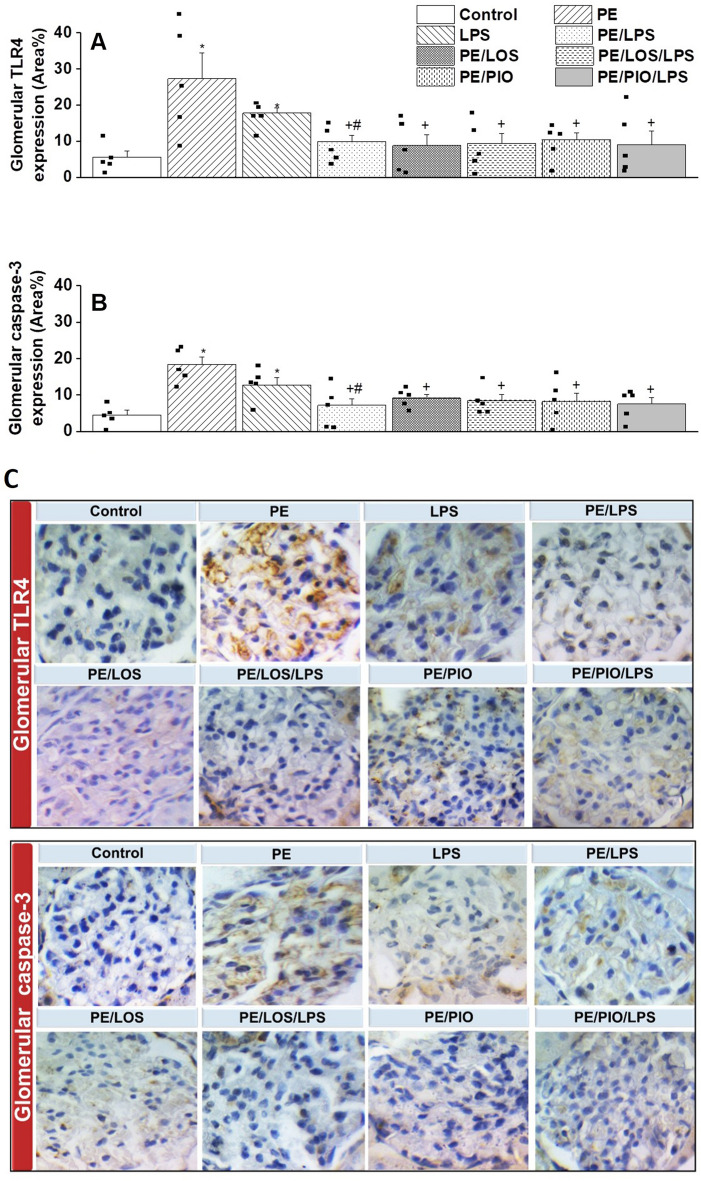
Effect of gestational losartan and pioglitazone therapy on immunohistochemical protein expressions of TLR-4 receptors (panel A) and Caspase-3 (panel B) in renal glomerular tissues of PE or PE/LPS dams. Representative images for immunostained renal glomerular tissues are shown in panel C. The One-way ANOVA followed by the Tukey’s post hoc was utilized to measure statistical significance. Values are expressed as means ± S.E.M of 5 observations. *p < 0.05 vs. “Control”, ^+^p < 0.05 vs. “PE and ^#^p < 0.05 vs. “LPS” values.

**Fig. 6:**
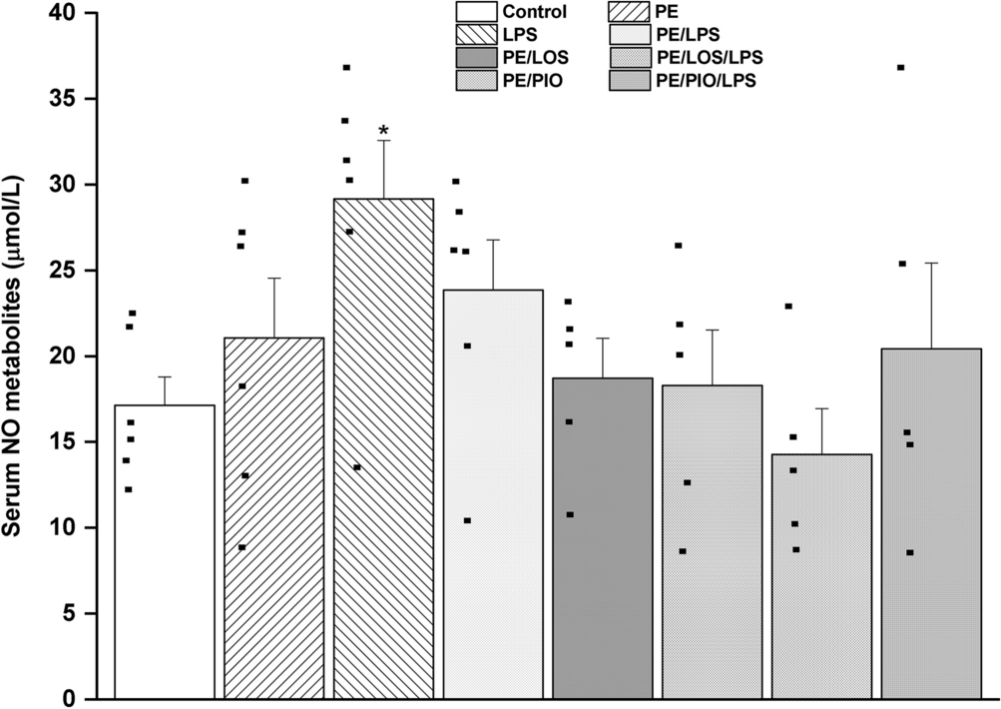
Effect of gestational losartan and pioglitazone therapy on serum nitric oxide metabolites of PE or PE/LPS dams measured colorimetry. The One-way ANOVA followed by the Tukey’s post hoc was utilized to measure statistical significance. Values are expressed as means ± S.E.M of 5–6 observations. *p < 0.05 vs. “Control”.

Immunohistochemical studies were performed to assess the possible involvement of the inflammatory TLR-4 and apoptotic caspase-3 signals in the renovascular PE/LPS interaction. The data in Fig. 4 showed that the protein expression of both TLR4 and Caspase-3 in tubular tissues were significantly augmented by PE (n = 5, p = 0.0096 and 0.0037, respectively) or LPS insult (n = 5, p = 0.0099 and 0.0099, respectively). Similar increases in these signaling molecules in response to PE (p = 0.0020 and 0.0002, respectively) or LPS (p = 0.0006 and 0.0109, respectively) were also observed in glomerular tissues (n = 5, Fig. 5). With the exception of tubular TLR4, these inflammatory and apoptotic signals incited by individual insults were mostly attenuated in dams exposed to the double PE/LPS challenge (Figs. 4–5, n = 5). Similarly, the PE-mediated rises in tubular (Figs. 4) and glomerular (Figs. 5) TLR4 and caspase-3 expression were neutralized upon prenatal exposure to losartan (n = 5) or pioglitazone (n = 5). Compared with control (non-PE) rats, serum levels of NO metabolites were significantly increased after LPS treatment (p = 0.0100) but remained unchanged in other rat groups (n = 5, Fig. 6).

### Effects of gestational losartan or pioglitazone on renal histopathological investigations

**Fig. 7:**
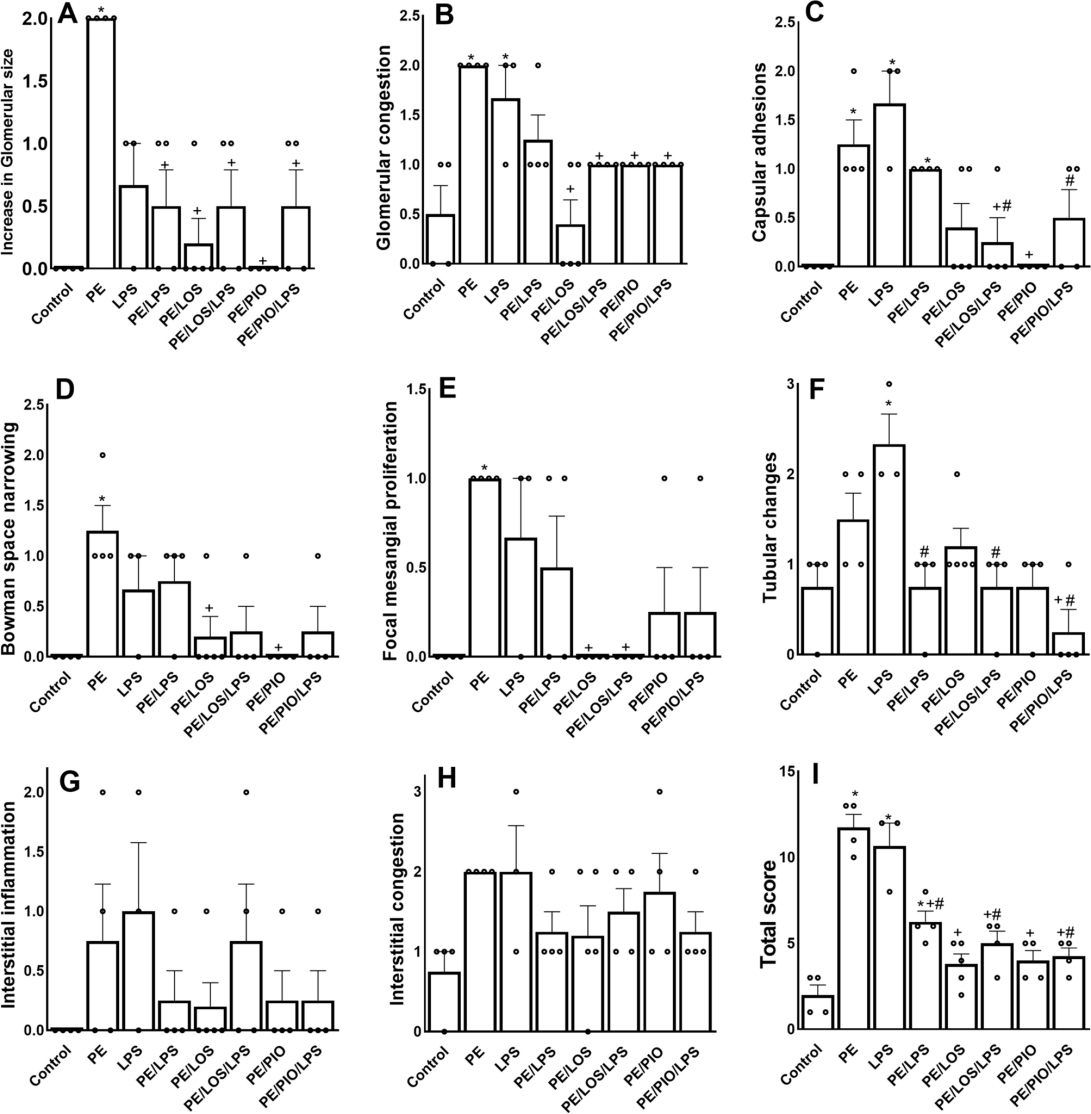
Effect of gestational losartan and pioglitazone therapy on renal histopathology, increase in glomerular size (panel A), glomerular capillary congestion (panel B), capsular adhesions (panel C), Bowman space narrowing (panel D), focal mesangial proliferation (panel E), tubular changes (panel F), interstitial inflammation (panel G), interstitial congestion (panel H) and total histopathology score (panel I) in tissues of PE or PE/LPS dams. Data are expressed as means ± SEM (n = 4). The One-way ANOVA followed by the Tukey’s post hoc was utilized to measure statistical significance. *p < 0.05 vs. “Control”, + p < 0.05 vs. “PE and #p < 0.05 vs. “LPS” values.

**Fig. 8:**
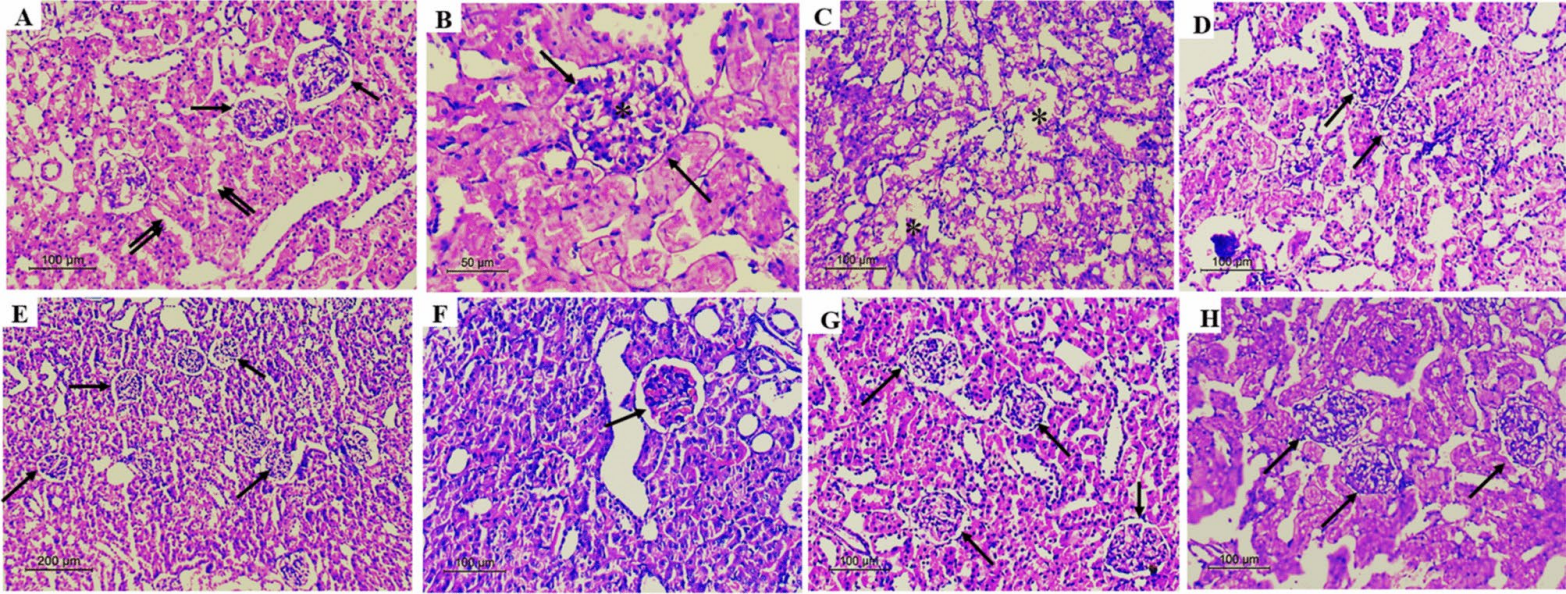
Descriptive photomicrographs of renal sections. (A) Control rat: Kidney showing intact glomeruli with normal cellularity and intact Bowman space (arrows), tubular lining epithelium is intact (double arrows) and there is no evidence of interstitial inflammation or congestion (H&Ex200). (B) PE: An enlarged glomerulus featuring hypercellularity (asterisk) with marked Bowman space narrowing (arrows) (H&Ex400). (C) LPS: There is an evident cloudy swelling in the tubular lining (asterisks) (H&Ex200). (D) PE/LPS: Normal-sized glomeruli with minimal glomerular capillary congestion (arrows) (H&Ex200). (E) PE/LOS: Kidney showing intact glomeruli with normal cellularity and intact Bowman space (arrows) (H&Ex100). (F) PE/LOS/LPS: A normal-sized glomerulus with an intact Bowman space without capsular adhesions (arrow) and the tubules appear intact (H&Ex200). (G) PE/ PIO: Kidney showing glomeruli with minimal glomerular capillary congestion and intact Bowman space (arrows) (H&Ex200). (H) PE/ PIO/LPS: Glomeruli showing minimal glomerular capillary congestion with Bowman space narrowing (arrows) and the tubules appear intact (H&Ex200).

Histopathological examination of H&E-stained renal sections by light microscopy in weaning rats showed that compared with non-PE renal tissues, PE kidneys exhibited a significant increase in glomerular size (Fig. 7A, p < 0.0001), glomerular capillary congestion (Fig. 7B, p = 0.0003), capsular adhesions (Fig. 7C, p = 0.0074), Bowman space narrowing (Fig. 7D, p = 0.0083) and focal mesangial proliferation (Fig. 7E, p = 0.0135). Alternatively, LPS significantly increased glomerular capillary congestion (Fig. 7B, p = 0.0122), capsular adhesions (Fig. 7C, p = 0.0007) and tubular changes appearing as cloudy swelling (Fig. 7F, p = 0.0088) only. No changes were noted in interstitial inflammation (Fig. 7G) or congestion (Fig. 7H). Morphological changes incited by PE or LPS insults were echoed by significant rises in the total histopathological score in Fig. 7I. Most of these morphological deformities improved following double PE/LPS challenge or prenatal exposure to losartan or pioglitazone. Representative photomicrographs of these morphological findings are depicted in Fig. 8

## Discussion

The current study investigated the reprogramming effect of prenatal exposure to losartan or pioglitazone on hemodynamic and renovascular consequences of the PE/LPS challenge. The data showed that gestational therapy with either drug nullified preeclamptic manifestations of hypertension, proteinuria, and impaired renal vasodilation in weaning PE dams. The PE-provoked expression of inflammatory (TLR4) and apoptotic (caspase-3) signals in renal glomerular and tubular tissues were also suppressed by losartan or pioglitazone. The data suggests a protective effect for losartan and pioglitazone against renovascular dysfunction and interrelated inflammatory and apoptotic insults induced by PE. We also report that challenging weaning PE dams with LPS increased renal vasodilation, intensified the upregulated vasodilatory response evoked by gestational losartan or pioglitazone, and suppressed TLR4/Caspase-3 expressions, indicating a conditioning effect for endotoxemia on PE-evoked renovascular complications.

Several preclinical models have been developed to induce symptoms reminiscent of PE phenotype and imitate the pathological changes observed in humans^51^. In the current investigation, the daily injection of the NOS inhibitor L-NAME into pregnant dams for 7 consecutive days increased SBP and proteinuria compared to control counterparts. This is consistent with previous studies in which gestational NOS inhibition replicated some hallmarks of PE^52,53^. Remarkably, nitric oxide has been recognized as an important mediator of hemodynamic adaptation during gestation^54^ and preservation of healthy glomerular filtration barrier^55^. The elevated proteinuria caused by gestational L-NAME dosing results possibly from the associated renal morphological damage as indicated by the current findings of glomerular hypertrophy, glomerular capillary congestion, Bowman space narrowing and focal mesangial proliferation. Remarkably, the role of nitric oxide synthase inhibition in glomerular damage and increased glomerular hydraulic pressure and permeability has been emphasized by others^55,56^. Moreover, the present observation of reduced renal vasodilatory response to ACh in weaning PE dams is in line with previous reports that highlighted remarkable reductions in cholinergic relaxation in different vascular beds of PE rats^57–59^. Unlike the diminished cholinergic response, renal vasodilation induced by the adenosine analogue NECA was preserved in PE preparations. This may be accounted for by discrepancies in the mechanisms by which the two vasodilators relax vascular smooth muscle. Whereas the ACh response is essentially mediated via the endothelium-derived nitric oxide^60^, adenosinergic vasodilation involves not only an endothelium-dependent component^61^ but also direct adenylate cyclase-mediated relaxation of vascular smooth muscle^62^. Even in studies in which the endothelium appears to mediate the adenosine-induced vasodilation, there are conflicting data regarding whether the response is mediated by nitric oxide^63^ or other endothelium-derived factors^64^.

Considering that PE^3^ and perinatal endotoxemia^11^ are pregnancy-related pathologies and that the incidence of sepsis, septic shock, and septic pelvic thrombophlebitis is enhanced in preeclamptic mothers^43,44^, we hypothesized that the simultaneous exposure to preeclamptic and septic insults would elicit more deteriorated renal and inflammatory profiles. Contrary to our expectation, the current data revealed that the treatment of weaning PE dams with LPS diminished the renal structural damage and improved the depressed cholinergically-mediated renal vasodilatory response and restored ACh vasodilations back to respective non-PE levels. Arguably, this may suggest a post-conditioning action for endotoxemia against the PE-associated renal damage. The phenomenon of pre- or post-insult conditioning has been credited for LPS against a variety of tissue insults. For example, post-injury treatment with LPS was found to reduce cell death in traumatic brain injury^65^ and infarct volume following middle cerebral artery occlusion^66^. Additionally, endotoxin preconditioning has been documented in curtailing renal damage^67,68^ and ischemic stroke^69^. Albeit, the advantageous post-conditioning action of LPS against preeclamptic renovascular perturbations in weaning mothers is at odds with our previous reports in which LPS intensifies cardiac autonomic neuropathy in weaning PE mothers and offspring^70,71^. Speculatively, this implies that the outcome of the PE/LPS interaction is organ and/or function specific.

A hostile role has been proposed for RAS in the pathophysiology of PE^27,28^ and sepsis^31,32^. The negative pharmacologic manipulation of RAS by interventions like RAS inhibitors or PPAR-γ activators acts favorably to reconcile PE manifestations. For instance, Doering et al.^29^ and Zhou et al.^30^ showed that AT1 receptor blockade by losartan rectifies signs of the PE phenotype including hypertension, proteinuria and reduced litter weights. Similar beneficial effects have been observed after PPAR-γ activation^48,50^. These findings prompted us to investigate the effects of gestational AT1 receptor blockade or PPAR-γ stimulation by losartan and pioglitazone, respectively, on the PE/LPS interaction. The data showed several favorable effects for gestational losartan and pioglitazone therapies as exemplified by (i) diminution of the PE-evoked renal structural damage and proteinuria, (ii) reductions in the hypertensive response to PE with restoration of blood pressure to near-control (non-PE) values at weaning time, and (iii) significant rises in cholinergic (ACh) and adenosinergic (NECA) renal vasodilatory responses. Data of the renovascular studies are in accordance with previous studies that showed an improved endothelium-dependent dilation in disease conditions following losartan^72^ or pioglitazone therapy^73^. Arguably, the use of AT1 receptor blockers during gestation is often disputed due to their teratogenic risk^74^. Furthermore, there is scarce information available about the safety of PPAR agonists such as pioglitazone during pregnancy^75^. However, the use of drugs such as losartan and pioglitazone as experimental tools could shed light on the interplay between AT1 receptors and PPAR-γ pathways in the pathophysiology of PE, which may lead to therapeutic insights for reprogramming PE-induced renal defects.

The likelihood of the advantageous losartan or pioglitazone effect on preeclamptic renovascular dysfunction receives more support and perhaps mechanistic insight from molecular studies, which measured the protein expression of inflammatory (TLR4) and apoptotic (caspase-3) markers in renal tissues. TLR4 was particularly targeted because it is a pattern recognition receptor that dysregulates the immune response and incites inflammation in pathologies like endotoxemia^24^ and PE^76^. Additionally, the inflammatory response provoked by TLR4 activation upregulates caspase-3 and other apoptotic signals that play a central role in programmed cell death and organ dysfunction. Caspase-3 as such can enhance the production of inflammatory molecules, indicating a mutual facilitatory interplay between apoptotic and inflammatory pathways^77^. Immunohistochemical studies of the present study revealed two important observations. First, remarkably higher expression levels of TLR4 and caspase-3 in renal glomerular and tubular tissues of weaning PE dams compared with their control counterparts (Figs. 4 and 5). Second, the upregulated levels of these inflammatory and apoptotic signals disappeared in PE rats treated prenatally with losartan or pioglitazone. The downregulatory effect of these two therapies on TLR-4^78,79^ and caspase-3^80,81^ expression has also been reported in other models of renal injury. Notably, the precise cellular and molecular mechanisms of the interaction of PE with LPS on the one hand, and losartan or pioglitazone on the other hand, remain to be investigated.

Immunohistochemical studies also showed paradoxical effects for LPS on TLR4 and caspase-3 expressions in renal tissues of weaning PE (downregulation) and non-PE dams (upregulation). The observation that the inciting effect of PE on renal TLR4/caspase-3 abundance was mostly nullified by LPS provides a molecular basis for the counteracting effect of LPS on preeclamptic abnormalities in renovascular reactivity. The paradoxical effects of acute LPS exposure on renal TLR4/caspase-3 phenotype in non-PE (upregulation) and PE dams (downregulation) are consistent with the conditional action of LPS against inflammatory and apoptotic signals provoked by a variety of biological insults. For instance, Piao et al^82^ implicated the suppressed TLR4-triggered MyD88- and TRIF-dependent signaling pathways and accentuated expression of negative regulators of TLR like suppressor of IkappaB kinase-epsilon in the preconditioning action of LPS in human monocytes in endotoxic tolerance. Li et al^83^ demonstrated that neuroprotection against spinal cord injury evoked by LPS is triggered by the diminution of the expressions of apoptotic markers (caspase-3, cleaved caspase-3, and Bax) and augmentation of the expression of antiapoptotic marker Bcl-2. He et al.^68^ suggested that the upregulation of heat shock protein 27 and associated depression of oxidative and apoptotic bursts is another mechanism that arbitrates the renoprotective action of LPS against functional and structural renal deficits caused by ischemia/reperfusion injury. Others found that microRNA-146a mediates the negative regulatory action of LPS on the inflammatory response to kidney ischemia/reperfusion injury in rodents^67^. In this latter study, microRNA-146a was found to suppress NF-κB activation via the inhibition of IL-1 receptor-associated kinase and TNF receptor-associated factor 6. Notably, microRNAs are small non-coding molecules that that are critically involved in gene regulation and consequent mRNA degradation or translational repression^84^.

The LPS augmentation of renal vasodilations in PE rats deserves a comment. We were prompted to look at this effect of LPS as a favorable action because of two reasons. First, LPS augmented the depressed renal vasodilations evoked by PE but failed to do so when tested in non-PE preparations, suggesting that the LPS enhancement of renal vasodilations is specific to the PE state and its associated renovascular dysfunction. Second, in doing so, LPS replicated the beneficial renovascular influences of therapies like losartan and pioglitazone in PE mothers as clearly demonstrated in Figs. 2 and 3. Given that systemic vasodilation, hypovolemia, and reduced tissue perfusion are major cardiovascular complications of endotoxemia and endotoxic shock^85,86^, caution should be taken when framing the changes caused by LPS in renovascular function as a positive outcome.

In conclusion, this study reveals potential therapeutic effects for gestational pharmacologic therapies like losartan and pioglitazone on impaired renal vasodilatory responses to cholinergic and adenosinergic stimuli in weaning PE dams. The abolition of renal morphological defects evoked by PE and concomitant surges in renal inflammatory and apoptotic insults appear to mediate the beneficial actions of either drug. Renal aberrations induced by PE are also remarkably improved after postnatal endotoxic challenge with LPS. More studies are warranted to uncover more mechanistic insight into these interactions and ascertain its clinical relevance.

## Materials and methods

### Animals

Adult female Wistar rats (180–240 g) were obtained from the Animal facility of the Faculty of Pharmacy, Alexandria University, Egypt, maintained under controlled laboratory conditions and allowed free access to standard rat chow and tap water. All experimental protocols and animal manipulations were approved by the Institutional Animal Care and Use Committee, Alexandria University, Egypt (ACUC project, Approval No. AU06201957149, approval date. 7/5/2019), and in accordance with the "Principles of laboratory animal care" (NIH publication No. 86–23, revised 1985) and the ARRIVE guidelines. All methods were performed in accordance with the relevant guidelines and regulations.

### PE induction

Pregnancy was achieved by allowing the mating between nulliparous adult virgin female rats and larger male rats at a ratio 1:1. The day of conception was determined by the presence of a vaginal plug or spermatozoa in vaginal lavage. For the induction of PE, N^ω^-nitro-L-arginine methyl ester (nitric oxide synthase inhibitor, L-NAME) (50 mg/kg/day; Sigma-Aldrich Co, St. Louis, MO, USA) was administered via oral gavage for 7 consecutive days starting from day 14 of conception^45,46^. The measurements of systolic blood pressure (SBP) by the tail-cuff technique (see below) as well as urinary protein level were used to validate PE development. At weaning, rats were processed for hemodynamic and renovascular studies.

### The rat isolated perfused kidney

The isolated perfused kidney technique was carried out to assess renal vasodilator capacities to ACh and NECA according to the method described in previous studies^87–89^. After the induction of anesthesia with i.p. thiopental (50 mg/kg; Biochemie, Vienna, Austria), the left kidney was exposed through a midline ventral laparotomy and the left renal artery was cannulated. The left kidney was then excised from surrounding tissue, rapidly mounted on a temperature-controlled glass chamber maintained at 37°C, continuously perfused with Krebs’ solution (NaCl 120, KCl 5, CaCl_2_ 2.5, MgSO_4_.7H_2_O 1.2, KH_2_PO_4_ 1.2, NaHCO_3_ 25, and glucose 11 mM), maintained at 37°C and gassed with 95% O_2_ and 5% CO_2_. The kidney was perfused at a constant flow rate of 5 ml/min by the means of a peristaltic pump (Model P3- Pharmacia Fine Chemicals). The perfusion pressure was continuously monitored by means of a BP transducer (Model P23XL, Astro-Med, Inc., West Warwick, RI, USA) connected to a computerized data acquisition system with LabChart-7 pro software (AD Instruments, Bella Vista, Australia). After a stabilization period of at least 30 min, the renal perfusion pressure was elevated by continuous infusion with the α_1_-adrenoceptor agonist phenylephrine (20 μM; Sigma-Aldrich Co, St. Louis, MO, USA). Cumulative dose response curves to bolus injections of ACh (0.01–7.29 nmol; Sigma-Aldrich Co, St. Louis, MO, USA) and NECA (1.6–100 nmol ; Sigma-Aldrich Co, St. Louis, MO, USA) were established by direct injection into the perfusate line proximal to the kidney. ACh and NECA were prepared freshly in distilled water and dimethyl sulfoxide (Loba Chemie Pvt Ltd, India), respectively. Each dose of ACh or NECA (100 µl) was injected when the former dose has achieved its maximal vasodilatory response. Moreover, a wash period of 30 min was allowed in each kidney after the last dose of ACh before establishing the NECA curve.

### Tail-cuff plethysmography

Non-invasive SBP measurements for conscious rats were performed using the tail-cuff technique and a computerized data acquisition system with LabChart-7 pro software (Power Lab 4/30, model ML866/P, AD Instruments, Bella Vista, Australia)^90,91^. All rats were subjected to daily pre-conditioning for at least 3 consecutive days before the actual measurement of SBP to get the rats adapted to measurement conditions. A rat specific tail cuff and pulse transducer (Pan Lab, Spain) were placed on the base of the tail. SBP measurement was carried out based on the periodic occlusion of tail blood flow. SBP was measured in triplicates and values were averaged.

### Immunohistochemistry

The method described in our previous studies^81,92^ was employed to measure the expression of the inflammatory TLR-4 and apoptotic signals (caspase-3) in glomerular tissues as well as in outer medullary areas of tubular cortex. Sections (4 μm thick) of kidney were deparaffinized in xylene and rehydrated in a series of declining ethanol concentrations (100, 95 and 70%). Heat-induced epitope retrieval was carried out by immersing slides in coplin jars containing 10 mM citrate buffer solution and incubated in a microwave at power 100 for 1 min then power 30 for 9 min. Endogenous peroxidases were blocked by 0.3% hydrogen peroxide for 10 min. The rabbit, anti-rat primary antibodies, caspase-3 (1μg/μl, Thermo Scientific®, Berlin, Germany, and TLR-4 (0.25 μg/μl, Thermo Scientific®) were diluted (1:300), applied to the slides and then sections were incubated at 4 °C overnight. The secondary antibody (HRP conjugate) was applied for 30 min. The chromogen 3, 3′-diaminobenzidine was prepared and applied as instructed by the manufacturer for protein visualization. Slides were counterstained with hematoxylin and dipped in ascending concentrations of alcohol and then xylene. For each section, not less than 10 images were taken using Optika® Optikam B9 digital camera mounted on Optika® B-193 microscope using the company’s Vision Lite software version 2.13. The mean of these images represented the result of the section. Fiji Image J Software Version 1.53c (National Institutes of Health, Bethesda, Maryland, USA) was used to measure the percentage of chromogen 3,3′-diaminobenzidine positive stained area.

### Urine analyses

On gestational day 20, pregnant rats were kept in metabolic cages with mesh wire bottom made of stainless steel and allowed access to standard rat chow and water. The 24-h urine samples were collected under light mineral oil and stored at -80 °C until processed^71^. Urinary protein levels were measured by the pyrogallol red method^93^ using Cromatest standard kit (LiNEAR Chemicals, Spain) according to the manufacturer’s guidelines.

### Serum analyses

Retro-orbital blood samples were withdrawn from thiopental (50 mg/kg i.p.) anesthetized rats prior to kidney isolation. The collected blood was permitted to coagulate for 15 min at room temperature and centrifuged at 1200 *g* for 10 min. The aspirated serum was stored at -80 °C for subsequent colorimetric determination of NO metabolites according to the manufacturer’s instructions (BioDiagnostic, Giza, Egypt).

### Histopathological investigations

For histological analysis, kidney tissues were fixed in 10% neutral-buffered formalin and embedded in paraffin, sectioned at 5 μm intervals and stained with haematoxylin and eosin (H&E). Light microscopy was used to determine the morphologic changes. Scoring of each histopathological feature was performed as described in our previous study^81^ using the following arbitrary scale: 0 = absent; 1 =  < 10%; 2 = 11–24%; 3 = 25–50% of surface area examined.

## Protocols and Experimental Groups

### Preeclamptic maternal programming of renal vasodilatory and hemodynamic sequels of endotoxemia

This experiment investigated the programming effect of PE on hemodynamic and renal vasodilator manifestations of endotoxemia in weaning mothers. Three weeks after spontaneous delivery, a total of 4 groups of weaning mothers (n = 6–8 each) were employed in the current study and assigned as follows: (i) saline-treated non-PE mothers, (ii) saline-treated PE mothers, (iii) LPS-treated non-PE mothers, and (iv) LPS-treated PE mothers. Endotoxemia was induced by i.p. administration of a 5 mg/kg dose of LPS (from E coli, serotype 0111:B4; Sigma-Aldrich Co, St. Louis, MO, USA) in weaning mothers^18^. LPS was dissolved in saline. Four hours later, SBP was measured by the tail-cuff technique, rats were anesthetized with thiopental (50 mg/kg i.p), and left kidneys were isolated and perfused for the assessment of renal vasodilator responsiveness to ACh (0.01–7.29 nmol) and NECA (1.6–100 nmol). The right kidneys were collected for immunohistochemical determination of TLR-4 and caspase-3 expression and histopathological investigations in renal tissues.

### Impact of gestational losartan or pioglitazone administration on preeclamptic endotoxic manifestations

This experiment investigated the effect of gestational administration of losartan or pioglitazone on hemodynamic and renal vasodilator defects caused by PE or PE/LPS insult in weaning mothers three weeks postpartum. Following pregnancy induction, Losartan (10 mg/kg; PHARCO Pharmaceutical Co., Alexandria, Egypt) or pioglitazone (5 mg/kg; PHARCO Pharmaceutical Co., Alexandria, Egypt)^94–96^ were administered to other groups of pregnant rats along with L-NAME (50 mg/kg/day) for 7 consecutive days, starting from gestational day 14 till delivery. Three weeks after spontaneous delivery, four groups of weaning rats (n = 6–8) were employed in this study and assigned as follows: (i) saline-treated PE/losartan mothers, (ii) LPS-treated PE/losartan mothers, (iii) saline-treated PE/pioglitazone mothers, (iv) LPS-treated PE/pioglitazone mothers. Endotoxemia was induced by i.p. injection of a 5 mg/kg dose of LPS and 4 h later, rats were processed for renovascular, immunohistochemical and histopathological studies as described in the previous experiment.

## Statistics

Values are expressed as means ± S.E.M. The vasodilatory responses to ACh and NECA were expressed as the percentage of the precontraction level induced by 20 μM phenylephrine. The cumulative vasodilatory effects of ACh and NECA were computed by calculating the area under the curve (AUC) for individual experiments using trapezoidal integration and zero line as the baseline. In immunohistochemical studies, the percentages of stained areas were estimated. The one-way ANOVA followed by the Tukey’s post hoc test was used to assess statistical significance with probability levels < 0.05. Normal distribution was checked using column statistics (Shapiro–wilk normality test, GraphPad Prism, software release 8.0.2).

## Supplementary Information

Supplementary Information 1.

Supplementary Information 2.

Supplementary Information 3.

Supplementary Information 4.

## Data Availability

Raw data are provided as additional supporting files.

## References

[1] Rao, S. & Jim, B. Acute kidney injury in pregnancy: The changing landscape for the 21st century. *Kidney Int. rep.* **3**, 247–257. 10.1016/j.ekir.2018.01.011 (2018). 29725629 10.1016/j.ekir.2018.01.011 PMC5932309

[2] Sacks, G. P., Studena, K., Sargent, K. & Redman, C. W. Normal pregnancy and preeclampsia both produce inflammatory changes in peripheral blood leukocytes akin to those of sepsis. *Am. J. Obstet. Gynecol.* **179**, 80–86. 10.1016/s0002-9378(98)70254-6 (1998). 9704769 10.1016/s0002-9378(98)70254-6

[3] Karatza, A. A. & Dimitriou, G. Preeclampsia emerging as a novel risk factor for cardiovascular disease in the offspring. *Curr. Pediatr. Rev.* **16**, 194–199. 10.2174/1573396316666191224092405 (2020). 31884930 10.2174/1573396316666191224092405 PMC8193805

[4] Tranquilli, A. L. et al. The classification, diagnosis and management of the hypertensive disorders of pregnancy: A revised statement from the ISSHP. *Pregnancy hypertens.* **4**, 97–104. 10.1016/j.preghy.2014.02.001 (2014). 26104417 10.1016/j.preghy.2014.02.001

[5] Moghaddas Sani, H., Zununi Vahed, S. & Ardalan, M. Preeclampsia: A close look at renal dysfunction. *Biomed. Pharmacother.= Biomed. Pharmacother.* **109**, 408–416. 10.1016/j.biopha.2018.10.082 (2019). 30399576 10.1016/j.biopha.2018.10.082

[6] Fishel Bartal, M., Lindheimer, M. D. & Sibai, B. M. Proteinuria during pregnancy: Definition, pathophysiology, methodology, and clinical significance. *Am. J. Obstet. Gynecol.* **226**, S819–S834. 10.1016/j.ajog.2020.08.108 (2022). 32882208 10.1016/j.ajog.2020.08.108

[7] Harmon, A. C. et al. The role of inflammation in the pathology of preeclampsia. *Clin. Sci. (Lond. Engl.: 1979)* **130**, 409–419. 10.1042/cs20150702 (2016). 10.1042/CS20150702 PMC5484393 26846579

[8] Cornelius, D. C. Preeclampsia: From Inflammation to Immunoregulation. *Clin. Med. insights. Blood disord.*10.1177/1179545x17752325 (2018). 29371787 10.1177/1179545X17752325 PMC5772493

[9] Wedn, A. M., El-Bassossy, H. M., Eid, A. H. & El-Mas, M. M. Modulation of preeclampsia by the cholinergic anti-inflammatory pathway: Therapeutic perspectives. *Biochem. Pharmacol.* **192**, 114703. 10.1016/j.bcp.2021.114703 (2021). 34324867 10.1016/j.bcp.2021.114703

[10] Turbeville, H. R. & Sasser, J. M. Preeclampsia beyond pregnancy: Long-term consequences for mother and child. *Am. J. Physiol. Ren. Physiol.* **318**, F1315–F1326. 10.1152/ajprenal.00071.2020 (2020). 10.1152/ajprenal.00071.2020 PMC7311709 32249616

[11] Nayak, A. H. & Khade, S. A. Obstetric sepsis: A review article. *J. obstet. Gynaecol. India* **72**, 470–478. 10.1007/s13224-022-01706-y (2022). 36506893 10.1007/s13224-022-01706-y PMC9732161

[12] Einav, S. & Leone, M. Epidemiology of obstetric critical illness. *Int. J. obstet. Anesth.* **40**, 128–139. 10.1016/j.ijoa.2019.05.010 (2019). 31257034 10.1016/j.ijoa.2019.05.010

[13] Cai, L., Rodgers, E., Schoenmann, N. & Raju, R. P. Advances in rodent experimental models of sepsis. *Int. J. Mol. Sci.*10.3390/ijms24119578 (2023). 37298529 10.3390/ijms24119578 PMC10253762

[14] Bhor, V. M., Thomas, C. J., Surolia, N. & Surolia, A. Polymyxin B: An ode to an old antidote for endotoxic shock. *Mol. Biosyst.* **1**, 213–222. 10.1039/b500756a (2005). 16880985 10.1039/b500756a

[15] Zhang, S. et al. Interception of the endotoxin-induced arterial hyporeactivity to vasoconstrictors. *Vasc. Pharmacol.* **62**, 15–23. 10.1016/j.vph.2014.04.005 (2014). 10.1016/j.vph.2014.04.005 PMC4374743 24792896

[16] Chuang, C. H., Yang, C. K., Wu, P. H., Zhang, Y. & Yang, P. J. Acute renal injury induced by endotoxic shock in rats is alleviated via PI3K/Nrf2 pathway. *Eur. Rev. Med. Pharmacol. Sci.* **22**, 5394–5401. 10.26355/eurrev_201808_15742 (2018). 30178867 10.26355/eurrev_201808_15742

[17] Shen, H. H. et al. Alpha-lipoic acid prevents endotoxic shock and multiple organ dysfunction syndrome induced by endotoxemia in rats. *Shock (Augusta, Ga.)* **43**, 405–411. 10.1097/shk.0000000000000295 (2015). 25514429 10.1097/SHK.0000000000000295

[18] Wedn, A. M., El-Gowilly, S. M. & El-Mas, M. M. Time and sex dependency of hemodynamic, renal, and survivability effects of endotoxemia in rats. *Saudi pharma. J.: SPJ: off. Publ. Saudi Pharm. Soc.* **28**, 127–135. 10.1016/j.jsps.2019.11.014 (2020). 10.1016/j.jsps.2019.11.014 PMC6950976 31933528

[19] Mazgaeen, L. & Gurung, P. Recent advances in lipopolysaccharide recognition systems. *Int. J. Mol. Sci.*10.3390/ijms21020379 (2020). 31936182 10.3390/ijms21020379 PMC7013859

[20] Chousterman, B. G., Swirski, F. K. & Weber, G. F. Cytokine storm and sepsis disease pathogenesis. *Semin. Immunopathol.* **39**, 517–528. 10.1007/s00281-017-0639-8 (2017). 28555385 10.1007/s00281-017-0639-8

[21] Gómez, H. & Kellum, J. A. Sepsis-induced acute kidney injury. *Curr. Opin. Crit. Car.* **22**, 546–553. 10.1097/mcc.0000000000000356 (2016). 10.1097/MCC.0000000000000356 PMC5654474 27661757

[22] Jim, B. & Karumanchi, S. A. Preeclampsia: Pathogenesis, prevention, and long-term complications. *Semi. Nephrol.* **37**, 386–397. 10.1016/j.semnephrol.2017.05.011 (2017). 10.1016/j.semnephrol.2017.05.011 28711078

[23] Deer, E. et al. The role of immune cells and mediators in preeclampsia. *Nat. Rev. Nephrol.* **19**, 257–270. 10.1038/s41581-022-00670-0 (2023). 36635411 10.1038/s41581-022-00670-0 PMC10038936

[24] Li, Q. et al. Increased TLR4 expression aggravates sepsis by promoting IFN-γ expression in CD38(-/-) mice. *J. Immunol. Res.* **2019**, 3737890. 10.1155/2019/3737890 (2019). 30915370 10.1155/2019/3737890 PMC6399547

[25] Bernardi, F. C. et al. Oxidative damage, inflammation, and Toll-like receptor 4 pathway are increased in preeclamptic patients: A case-control study. *Oxid. Med. Cell. Longev.* **2012**, 636419. 10.1155/2012/636419 (2012). 22792416 10.1155/2012/636419 PMC3388586

[26] Clark, C. R. & Khalil, R. A. Regulation of vascular angiotensin II type 1 and type 2 receptor and angiotensin-(1–7)/MasR signaling in normal and hypertensive pregnancy. *Biochem. Pharmacol.* **220**, 115963. 10.1016/j.bcp.2023.115963 (2024). 38061417 10.1016/j.bcp.2023.115963 PMC10860599

[27] Leal, C. R. V. et al. Renin-angiotensin system in normal pregnancy and in preeclampsia: A comprehensive review. *Pregnancy hypertens.* **28**, 15–20. 10.1016/j.preghy.2022.01.011 (2022). 35149272 10.1016/j.preghy.2022.01.011

[28] Lumbers, E. R., Delforce, S. J., Arthurs, A. L. & Pringle, K. G. Causes and consequences of the dysregulated maternal renin-angiotensin system in preeclampsia. *Front. Endocrinol.* **10**, 563. 10.3389/fendo.2019.00563 (2019). 10.3389/fendo.2019.00563 PMC6746881 31551925

[29] Doering, T. P., Haller, N. A., Montgomery, M. A., Freeman, E. J. & Hopkins, M. P. The role of AT1 angiotensin receptor activation in the pathogenesis of preeclampsia. *Am. J. obstet. Gynecol.* **178**, 1307–1312. 10.1016/s0002-9378(98)70337-0 (1998). 9662316 10.1016/s0002-9378(98)70337-0

[30] Zhou, C. C. et al. Angiotensin receptor agonistic autoantibodies induce pre-eclampsia in pregnant mice. *Nat. Med.* **14**, 855–862. 10.1038/nm.1856 (2008). 18660815 10.1038/nm.1856 PMC3267158

[31] Senatore, F., Balakumar, P. & Jagadeesh, G. Dysregulation of the renin-angiotensin system in septic shock: Mechanistic insights and application of angiotensin II in clinical management. *Pharmacol. Res.* **174**, 105916. 10.1016/j.phrs.2021.105916 (2021). 34597810 10.1016/j.phrs.2021.105916

[32] Corrêa, T. D., Takala, J. & Jakob, S. M. Angiotensin II in septic shock. *Crit. care (Lond. Engl.)* **19**, 98. 10.1186/s13054-015-0802-3 (2015). 10.1186/s13054-015-0802-3 PMC4360936 25886853

[33] Rosa, R. M. et al. Alternative pathways for angiotensin II production as an important determinant of kidney damage in endotoxemia. *Am. J. Physiol. Ren. Physiol.* **311**, F496–F504. 10.1152/ajprenal.00121.2014 (2016). 10.1152/ajprenal.00121.2014 27252489

[34] Bucher, M., Ittner, K. P., Hobbhahn, J., Taeger, K. & Kurtz, A. Downregulation of angiotensin II type 1 receptors during sepsis. *Hypertens. (Dallas, Tex.: 1979)* **38**, 177–182. 10.1161/01.hyp.38.2.177 (2001). 10.1161/01.hyp.38.2.177 11509472

[35] Zhan, Z., Lian, Z. & Bai, H. Dexamethasone inhibited angiotensin II and its receptors to reduce sepsis-induced lung and kidney injury in rats. *PloS one* **19**, e0308557. 10.1371/journal.pone.0308557 (2024). 39178201 10.1371/journal.pone.0308557 PMC11343412

[36] Fukada, M. et al. Systemic administration of lipopolysaccharide upregulates angiotensin II expression in rat renal tubules: Immunohistochemical and ELISA studies. *Peptides* **26**, 2215–2221. 10.1016/j.peptides.2005.03.057 (2005). 15963602 10.1016/j.peptides.2005.03.057

[37] du Cheyron, D., Lesage, A., Daubin, C., Ramakers, M. & Charbonneau, P. Hyperreninemic hypoaldosteronism: A possible etiological factor of septic shock-induced acute renal failure. *Intens. care Med.* **29**, 1703–1709. 10.1007/s00134-003-1986-6 (2003). 10.1007/s00134-003-1986-6 14551679

[38] Lund, D. D., Brooks, R. M., Faraci, F. M. & Heistad, D. D. Role of angiotensin II in endothelial dysfunction induced by lipopolysaccharide in mice. *Am. J. physiol. Heart. Circ. Physiol.* **293**, H3726–H3731. 10.1152/ajpheart.01116.2007 (2007). 17965276 10.1152/ajpheart.01116.2007

[39] Laesser, M., Oi, Y., Ewert, S., Fändriks, L. & Aneman, A. The angiotensin II receptor blocker candesartan improves survival and mesenteric perfusion in an acute porcine endotoxin model. *Acta anaesthesiol. Scand.* **48**, 198–204. 10.1111/j.0001-5172.2004.00283.x (2004). 14995942 10.1111/j.0001-5172.2004.00283.x

[40] Hirano, Y. et al. (Pro)renin receptor blocker improves survival of rats with sepsis. *J. surg. Res.* **186**, 269–277. 10.1016/j.jss.2013.08.004 (2014). 24011922 10.1016/j.jss.2013.08.004

[41] Chappell, L. C., Cluver, C. A., Kingdom, J. & Tong, S. Pre-eclampsia. *Lancet (Lond. Engl.)* **398**, 341–354. 10.1016/s0140-6736(20)32335-7 (2021). 10.1016/S0140-6736(20)32335-7 34051884

[42] Cecconi, M., Evans, L., Levy, M. & Rhodes, A. Sepsis and septic shock. *Lancet (Lond. Engl.)* **392**, 75–87. 10.1016/s0140-6736(18)30696-2 (2018). 10.1016/S0140-6736(18)30696-2 29937192

[43] Acosta, C. D. et al. The continuum of maternal sepsis severity: incidence and risk factors in a population-based cohort study. *PloS one* **8**, e67175. 10.1371/journal.pone.0067175 (2013). 23843991 10.1371/journal.pone.0067175 PMC3699572

[44] Isler, C. M. et al. Septic pelvic thrombophlebitis and preeclampsia are related disorders. *Hypertens. Pregnancy* **23**, 121–127. 10.1081/prg-120029858 (2004). 15117606 10.1081/PRG-120029858

[45] Ali, M. A. et al. Gestational NSAIDs distinctly reprogram cardiac injury in preeclamptic rats: Roles of cyclooxygenase, apoptotic and autophagic trails. *Life sci.* **310**, 121130. 10.1016/j.lfs.2022.121130 (2022). 36309226 10.1016/j.lfs.2022.121130

[46] Pandhi, P., Saha, L. & Malhotra, S. Effect of oral magnesium supplementation on experimental pre-eclampsia induced by prolonged blockade of nitric oxide synthesis in pregnant rats. *Ind.J. exp. Boil.* **40**, 349–351 (2002). 12635709

[47] Kvandova, M. et al. The peroxisome proliferator-activated receptor gamma agonist pioglitazone improves nitric oxide availability, renin-angiotensin system and aberrant redox regulation in the kidney of pre-hypertensive rats. *J. Physiol. Pharmacol.: An off. J. Polish Physiol. Soc.*10.26402/jpp.2018.2.09 (2018). 10.26402/jpp.2018.2.09 29980143

[48] Allam, H. I. G. & Masri, A. The potential therapeutic role of peroxisome ProliferatorActivated receptors agonist in Preeclamptic pregnant rats. *J. Coll. Phys. Surg. Pak.* **28**, 31–35. 10.29271/jcpsp.2018.01.31 (2018). 10.29271/jcpsp.2018.01.31 29290188

[49] Lane, S. L. et al. Pharmacological activation of peroxisome proliferator-activated receptor γ (PPAR-γ) protects against hypoxia-associated fetal growth restriction. *FASEB J.* **33**, 8999–9007. 10.1096/fj.201900214R (2019). 31039323 10.1096/fj.201900214R PMC6662983

[50] McCarthy, F. P. et al. Peroxisome proliferator-activated receptor-γ as a potential therapeutic target in the treatment of preeclampsia. *Hypertens. (Dallas, Tex.: 1979)* **58**, 280–286. 10.1161/HYPERTENSIONAHA.111.172627 (2011). 10.1161/HYPERTENSIONAHA.111.172627 21690483

[51] Mallette, J. H., Crudup, B. F. & Alexander, B. T. Growth Restriction in Preeclampsia: Lessons from Animal Models. *Curr. Opin. Physiol.*10.1016/j.cophys.2023.100647 (2023). 36968132 10.1016/j.cophys.2023.100647 PMC10035651

[52] Chen, Y. et al. Ferulic acid ameliorated placental inflammation and apoptosis in rat with preeclampsia. *Clin. Exp. Hypertens. (New York, N.Y.: 1993)* **41**(524), 530. 10.1080/10641963.2018.1516773 (2019). 10.1080/10641963.2018.1516773 30183401

[53] Molnár, M., Sütö, T., Tóth, T. & Hertelendy, F. Prolonged blockade of nitric oxide synthesis in gravid rats produces sustained hypertension, proteinuria, thrombocytopenia, and intrauterine growth retardation. *Am. J. obstet. Gynecol.* **170**, 1458–1466. 10.1016/s0002-9378(94)70179-2 (1994). 7909994 10.1016/s0002-9378(94)70179-2

[54] Li, J., LaMarca, B. & Reckelhoff, J. F. A model of preeclampsia in rats: the reduced uterine perfusion pressure (RUPP) model. *Am. J. physiol. Heart Circ. Physiol.* **303**, H1–H8. 10.1152/ajpheart.00117.2012 (2012). 22523250 10.1152/ajpheart.00117.2012 PMC3404644

[55] Dolinina, J., Sverrisson, K., Rippe, A., Öberg, C. M. & Rippe, B. Nitric oxide synthase inhibition causes acute increases in glomerular permeability in vivo, dependent upon reactive oxygen species. *Am. J. Physiol. Ren. Physiol.* **311**, F984–F990. 10.1152/ajprenal.00152.2016 (2016). 10.1152/ajprenal.00152.2016 27681559

[56] Sharma, M., McCarthy, E. T., Savin, V. J. & Lianos, E. A. Nitric oxide preserves the glomerular protein permeability barrier by antagonizing superoxide. *Kidney Int.* **68**, 2735–2744. 10.1111/j.1523-1755.2005.00744.x (2005). 16316348 10.1111/j.1523-1755.2005.00744.x

[57] Crews, J. K., Herrington, J. N., Granger, J. P. & Khalil, R. A. Decreased endothelium-dependent vascular relaxation during reduction of uterine perfusion pressure in pregnant rat. *Hypertens. (Dallas, Tex.: 1979)* **35**, 367–372. 10.1161/01.hyp.35.1.367 (2000). 10.1161/01.hyp.35.1.367 10642326

[58] Zhu, M., Ren, Z., Possomato-Vieira, J. S. & Khalil, R. A. Restoring placental growth factor-soluble fms-like tyrosine kinase-1 balance reverses vascular hyper-reactivity and hypertension in pregnancy. *Am. J. physiol. Regul. Integr. Comp. Physiol.* **311**, R505–R521. 10.1152/ajpregu.00137.2016 (2016). 27280428 10.1152/ajpregu.00137.2016 PMC5142222

[59] Walsh, S. K., English, F. A., Johns, E. J. & Kenny, L. C. Plasma-mediated vascular dysfunction in the reduced uterine perfusion pressure model of preeclampsia: A microvascular characterization. *Hypertens. (Dallas, Tex.: 1979)* **54**(345), 351. 10.1161/hypertensionaha.109.132191 (2009). 10.1161/HYPERTENSIONAHA.109.132191 19564546

[60] Matsumoto, T., Osada, T., Taguchi, K. & Kobayashi, T. Endothelial dysfunction in superior mesenteric arteries isolated from adenine-induced renal failure in model rats. *Biol. Pharm. Bull.* **46**, 1156–1160. 10.1248/bpb.b23-00234 (2023). 37532565 10.1248/bpb.b23-00234

[61] Fahim, M., el Mas, M. M., Abdel-Rahman, A. A. & Mustafa, S. J. Influence of aortic baroreceptor denervation on adenosine receptor-mediated relaxation of isolated rat aorta. *Eur. J. pharmacol.* **254**, 183–191. 10.1016/0014-2999(94)90386-7 (1994). 8206113 10.1016/0014-2999(94)90386-7

[62] Hourani, S. M., Boon, K., Fooks, H. M. & Prentice, D. J. Role of cyclic nucleotides in vasodilations of the rat thoracic aorta induced by adenosine analogues. *Br. J. Pharmacol.* **133**, 833–840. 10.1038/sj.bjp.0704140 (2001). 11454656 10.1038/sj.bjp.0704140 PMC1572848

[63] Smits, P. et al. Endothelial release of nitric oxide contributes to the vasodilator effect of adenosine in humans. *Circulation* **92**, 2135–2141. 10.1161/01.cir.92.8.2135 (1995). 7554193 10.1161/01.cir.92.8.2135

[64] Headrick, J. P. & Berne, R. M. Endothelium-dependent and -independent relaxations to adenosine in guinea pig aorta. *Am. J. physiol.* **259**, H62–H67. 10.1152/ajpheart.1990.259.1.H62 (1990). 2375414 10.1152/ajpheart.1990.259.1.H62

[65] Bingham, D., John, C. M., Panter, S. S. & Jarvis, G. A. Post-injury treatment with lipopolysaccharide or lipooligosaccharide protects rat neuronal and glial cell cultures. *Brain res. Bull.* **85**, 403–409. 10.1016/j.brainresbull.2011.04.007 (2011). 21571046 10.1016/j.brainresbull.2011.04.007

[66] Sardari, M. et al. Dose-dependent microglial and astrocytic responses associated with post-ischemic neuroprotection after lipopolysaccharide-induced sepsis-like state in mice. *Front. Cell. Neurosci.* **14**, 26. 10.3389/fncel.2020.00026 (2020). 32116567 10.3389/fncel.2020.00026 PMC7029732

[67] Dai, Y. et al. miR-146a is essential for lipopolysaccharide (LPS)-induced cross-tolerance against kidney ischemia/reperfusion injury in mice. *Sci. Rep.* **6**, 1–12. 10.1038/srep27091 (2016). 27250735 10.1038/srep27091 PMC4890025

[68] He, K., Xia, L. & Zhang, J. LPS ameliorates renal ischemia/reperfusion injury via Hsp27 up-regulation. *Int. Urol. Nephrol.* **50**, 571–580. 10.1007/s11255-017-1735-3 (2018). 29124510 10.1007/s11255-017-1735-3

[69] Vartanian, K. B. et al. LPS preconditioning redirects TLR signaling following stroke: TRIF-IRF3 plays a seminal role in mediating tolerance to ischemic injury. *J. Neuroinflamm.* **8**, 140. 10.1186/1742-2094-8-140 (2011). 10.1186/1742-2094-8-140 PMC3217906 21999375

[70] Abuiessa, S. A., El-Gowilly, S. M., El-Gowelli, H. M., Helmy, M. M. & El-Mas, M. M. Short-lived sensitization of cardiovascular outcomes of postpartum endotoxemia in preeclamptic rats: Role of medullary solitary tract neuroinflammation. *Eur. J. pharmacol.* **910**, 174494. 10.1016/j.ejphar.2021.174494 (2021). 34508754 10.1016/j.ejphar.2021.174494

[71] Abuiessa, S. A., Wedn, A. M., El-Gowilly, S. M., Helmy, M. M. & El-Mas, M. M. Pre-eclamptic fetal programming alters neuroinflammatory and cardiovascular consequences of endotoxemia in sex-specific manners. *J. pharmacol. Ex. Ther.* **373**, 325–336. 10.1124/jpet.119.264192 (2020). 10.1124/jpet.119.264192 32094295

[72] Cheetham, C. et al. Losartan, an angiotensin type 1 receptor antagonist, improves endothelial function in non-insulin-dependent diabetes. *J. Am. Coll. Cardiol.* **36**, 1461–1466. 10.1016/s0735-1097(00)00933-5 (2000). 11079643 10.1016/s0735-1097(00)00933-5

[73] El-Mas, M. M. et al. Pioglitazone abrogates cyclosporine-evoked hypertension via rectifying abnormalities in vascular endothelial function. *Biochem. Pharmacol.* **81**, 526–533. 10.1016/j.bcp.2010.11.013 (2011). 21114962 10.1016/j.bcp.2010.11.013 PMC3777403

[74] Alwan, S., Polifka, J. E. & Friedman, J. M. Angiotensin II receptor antagonist treatment during pregnancy Birth Defects Research Part A: Clinical and Molecular. *Teratology* **73**, 123–130. 10.1002/bdra.20102 (2005). 10.1002/bdra.20102 15669052

[75] Kung, J. & Henry, R. R. Thiazolidinedione safety. *Expert. Opin. Drug saf.* **11**, 565–579. 10.1517/14740338.2012.691963 (2012). 22616948 10.1517/14740338.2012.691963

[76] Pineda, A., Verdin-Terán, S. L., Camacho, A. & Moreno-Fierros, L. Expression of toll-like receptor TLR-2, TLR-3, TLR-4 and TLR-9 is increased in placentas from patients with preeclampsia. *Arch. Med. Res.* **42**, 382–391. 10.1016/j.arcmed.2011.08.003 (2011). 21843566 10.1016/j.arcmed.2011.08.003

[77] Wu, Z. et al. Programmed cell death in sepsis associated acute kidney injury. *Front. Med.* **9**, 883028. 10.3389/fmed.2022.883028 (2022). 10.3389/fmed.2022.883028 PMC9152147 35655858

[78] Tang, T. F., Zhou, Q. L., Zhu, L. L., Tang, R. & Ao, X. Effects of fosinopril and losartan on the expression of Toll- like receptor 4 in renal tubular epithelia cells. *Zhong nan da xue xue bao. Yi xue ban = J. Cent. South Univ. Med. Sci.* **33**(958), 965 (2008). 19001741

[79] Yu, Y., Wu, Y., Wen, G. & Yang, W. Effect of pioglitazone on the expression of TLR4 in renal tissue of diabetic rats. *Xi bao yu fen zi mian yi xue za zhi = Chin. J. cell. Mol. Immunol.* **30**, 793–797 (2014). 25108428

[80] Al-Kadi, A., El-Daly, M., El-Tahawy, N. F. G., Khalifa, M. M. A. & Ahmed, A. F. Angiotensin aldosterone inhibitors improve survival and ameliorate kidney injury induced by sepsis through suppression of inflammation and apoptosis. *Fund. Clin. Pharmacol.* **36**, 286–295. 10.1111/fcp.12718 (2022). 10.1111/fcp.12718 34309069

[81] Helmy, M. M., Helmy, M. W. & El-Mas, M. M. Additive renoprotection by pioglitazone and fenofibrate against inflammatory, oxidative and apoptotic manifestations of cisplatin nephrotoxicity: modulation by PPARs. *PloS one* **10**, e0142303. 10.1371/journal.pone.0142303 (2015). 26536032 10.1371/journal.pone.0142303 PMC4633146

[82] Piao, W. et al. Endotoxin tolerance dysregulates MyD88- and Toll/IL-1R domain-containing adapter inducing IFN-beta-dependent pathways and increases expression of negative regulators of TLR signaling. *J. leukoc. Boil.* **86**, 863–875. 10.1189/jlb.0309189 (2009). 10.1189/jlb.0309189 PMC2796624 19656901

[83] Li, W. C. et al. Lipopolysaccharide preconditioning attenuates apoptotic processes and improves neuropathologic changes after spinal cord injury in rats. *Int. j. neurosci.* **124**, 585–592. 10.3109/00207454.2013.864289 (2014). 24205811 10.3109/00207454.2013.864289

[84] Aghaei, S. M. & Hosseini, S. M. Inflammation-related miRNAs in obesity, CVD, and NAFLD. *Cytokine* **182**, 156724. 10.1016/j.cyto.2024.156724 (2024). 39106574 10.1016/j.cyto.2024.156724

[85] Cinel, I., Kasapoglu, U. S., Gul, F. & Dellinger, R. P. The initial resuscitation of septic shock. *J. crit. care* **57**, 108–117. 10.1016/j.jcrc.2020.02.004 (2020). 32135409 10.1016/j.jcrc.2020.02.004

[86] El-Lakany, M. A., Wedn, A. M. & El-Mas, M. M. In *Oxidative Stress in Cardiovascular-Metabolic Diseases* 227–296 (Springer, 2024).

[87] El-Gowelli, H. M., El-Gowilly, S. M., Elsalakawy, L. K. & El-Mas, M. M. Nitric oxide synthase/K+ channel cascade triggers the adenosine A(2B) receptor-sensitive renal vasodilation in female rats. *Eur. J. pharmacol.* **702**, 116–125. 10.1016/j.ejphar.2013.01.049 (2013). 23396225 10.1016/j.ejphar.2013.01.049

[88] Böke, T. & Malik, K. U. Enhancement by locally generated angiotensin II of release of the adrenergic transmitter in the isolated rat kidney. *J. Pharmacol. Exp.Ther.* **226**, 900–907 (1983). 6136604

[89] El-Mas, M. M., El-Gowilly, S. M., Gohar, E. Y. & Ghazal, A. R. Pharmacological characterization of cellular mechanisms of the renal vasodilatory effect of nicotine in rats. *Eur. J. Pharmacol.* **588**, 294–300. 10.1016/j.ejphar.2008.04.048 (2008). 18533147 10.1016/j.ejphar.2008.04.048

[90] El-Mas, M. M., Helmy, M. W., Ali, R. M. & El-Gowelli, H. M. Celecoxib, but not indomethacin, ameliorates the hypertensive and perivascular fibrotic actions of cyclosporine in rats: Role of endothelin signaling. *Toxicol. Appl. Pharmacol.* **284**, 1–7. 10.1016/j.taap.2015.01.018 (2015). 25656942 10.1016/j.taap.2015.01.018

[91] Pfeffer, J. M., Pfeffer, M. A. & Frohlich, E. D. Validity of an indirect tail-cuff method for determining systolic arterial pressure in unanesthetized normotensive and spontaneously hypertensive rats. *J. lab. Clin. Med.* **78**, 957–962 (1971). 5131859

[92] Sallam, M. Y., El-Gowilly, S. M. & El-Mas, M. M. Cardiac and brainstem neuroinflammatory pathways account for androgenic incitement of cardiovascular and autonomic manifestations in endotoxic male rats. *J. cardiovasc. Pharmacol.* **77**, 632–641. 10.1097/fjc.0000000000000993 (2021). 33852527 10.1097/FJC.0000000000000993

[93] Yalamati, P., Karra, M. L. & Bhongir, A. V. Comparison of urinary total proteins by four different methods. *Ind. J. clin. Biochem.: IJCB* **31**, 463–467. 10.1007/s12291-016-0551-3 (2016). 10.1007/s12291-016-0551-3 PMC4992489 27605745

[94] Abuiessa, S. A., Helmy, M. M., El-Gowelli, H. M., El-Gowilly, S. M. & El-Mas, M. M. Dysregulated ACE/Ang II/Ang1-7 signaling provokes cardiovascular and inflammatory sequelae of endotoxemia in weaning preeclamptic rats. *Eur. J. pharmacol.* **936**, 175344. 10.1016/j.ejphar.2022.175344 (2022). 36270538 10.1016/j.ejphar.2022.175344

[95] Ceravolo, G. S. et al. Enalapril and losartan restored blood pressure and vascular reactivity in intrauterine undernourished rats. *Life sci.* **80**, 782–787. 10.1016/j.lfs.2006.11.006 (2007). 17161436 10.1016/j.lfs.2006.11.006

[96] Ye, Y., Lin, Y., Perez-Polo, J. R. & Birnbaum, Y. Oral glyburide, but not glimepiride, blocks the infarct-size limiting effects of pioglitazone. *Cardiovasc. Drug. Ther.* **22**, 429–436. 10.1007/s10557-008-6138-3 (2008). 10.1007/s10557-008-6138-3 18825491

