# Supplementary material for: Pharmacologic and endotoxic reprogramming of renal vasodilatory, inflammatory, and apoptotic blemishes in weaning preeclamptic rats
Source: Sci Rep. 2025 Mar 8;15:8137. doi: 10.1038/s41598-025-87586-4 (PMC11890745; doi:10.1038/s41598-025-87586-4)
Supplement: Supplementary file 4 — Supplementary Information 4. [file 41598_2025_87586_MOESM4_ESM.docx]

6

| **i.s.**  **cong** | **i.s.**  **inflam** | **Tubular changes** | **Focal mesang prolif** | **B S narrowing** | **Adhesions** | **Glom**  **cong** | **Glom**  **size**↑ | **S** |
| --- | --- | --- | --- | --- | --- | --- | --- | --- |
|  | | | | | | | | |
|  |  |  |  |  |  |  |  | **Control** |
| 1 | 0 | 1 | 0 | 0 | 0 | 1 | 0 | 3w CM 2 |
| 0 | 0 | 1 | 0 | 0 | 0 | 0 | 0 | vc 3w CM 1 |
| 1 | 0 | 0 | 0 | 0 | 0 | 0 | 0 | vc 3w CM 5 |
| 1 | 0 | 1 | 0 | 0 | 0 | 1. | 0 | vc 3w CM 8 |
|  |  |  |  |  |  |  |  | **PE** |
| 2 | 1 | 1 | 1 | 1 | 1 | 2 | 2 | 3w Mo 8 |
| 2 | 2 | 2 | 1 | 1 | 1 | 2 | 2 | vc 3w Mo 6 |
| 2 | 0 | 1 | 1 | 1 | 1 | 2 | 2 | vc 3w Mo 7 |
| 2 | 0 | 2 | 1 | 2 | 2 | 2 | 2 | vc 3w Mo 9 |
|  |  |  |  |  |  |  |  | **LPS** |
| 1 | 2 | 2 | 0 | 0 | 1 | 2 | 0 | LPS 3w CM8 |
| 2 | 0 | 3 | 1 | 1 | 2 | 2 | 1 | vc LPS 3w CM5 |
| 3 | 1 | 2 | 1 | 1 | 2 | 1 | 1 | vc LPS 3w CM8 |
|  |  |  |  |  |  |  |  |  |
|  |  |  |  |  |  |  |  | **PE+LPS** |
| 1 | 1 | 1 | 1 | 1 | 1 | 2 | 0 | LPS 3w Mo2 |
| 2 | 0 | 1 | 0 | 0 | 1 | 1 | 0 | LPS 3w Mo3 |
| 1 | 0 | 1 | 0 | 1 | 1 | 1 | 1 | LPS 3w Mo7 |
| 1 | 0 | 0 | 1 | 1 | 1 | 1 | 1 | LPS 3w Mo8 |
|  |  |  |  |  |  |  |  | Los+PE |
| 0 | 1 | 1 | 0 | 0 | 1 | 0 | 0 | 3w 10 Mo CM |
| 1 | 0 | 1 | 0 | 0 | 0 | 0 | 0 | 3w 10 Mo 3 |
| 2 | 0 | 2 | 0 | 0 | 0 | 1 | 0 | 3w 10 Mo 6 |
| 1 | 0 | 1 | 0 | 1 | 0 | 1 | 1 | 3w 10 Mo 9 |
| 2 | 0 | 1 | 0 | 0 | 1 | 0 | 0 | 3w 10 Mo 10 |
|  |  |  |  |  |  |  |  | Los+PE+LPS |
| 1 | 0 | 1 | 0 | 1 | 1 | 1 | 1 | LPS 3w 0M02 |
| 2 | 2 | 1 | 0 | 0 | 0 | 1 | 0 | LPS 3w 10Mo1 |
| 1 | 0 | 0 | 0 | 0 | 0 | 1 | 1 | LPS 3w 10Mo3 |
| 2 | 1 | 1 | 0 | 0 | 0 | 1 | 0 | LPS 3w 10Mo4 |
|  |  |  |  |  |  |  |  | Pio+PE |
| 2 | 0 | 1 | 1 | 0 | 0 | 1 | 0 | 3w PM 01 |
| 1 | 0 | 1 | 0 | 0 | 0 | 1 | 0 | 3w PM 03 |
| 1 | 1 | 0 | 0 | 0 | 0 | 1 | 0 | 3w PM 05 |
| 3 | 0 | 1 | 0 | 0 | 0 | 1 | 0 | 3w PM 06 |
|  |  |  |  |  |  |  |  | Pio+PE+LPS |
| 1 | 1 | 0 | 0 | 0 | 0 | 1 | 0 | LPS 3w PM 2 |
| 1 | 0 | 0 | 1 | 0 | 1 | 1 | 1 | LPS 3w PM 0M |
| 2 | 0 | 0 | 0 | 1 | 0 | 1 | 1 | LPS 3w PM 01 |
| 1 | 0 | 1 | 0 | 0 | 1 | 1 | 0 | LPS 3w PM 06 |

* Scoring: 0=absent; 1 = <10%; 2 = 11-24%; 3 = 25-50% of surface area examined

**Glom Size: glomerular size**

**Glom Cong: glomerular capillary congestion**

**Adhesions: capsular adhesions**

**B S: Bowman space narrowing**

**Focal mesang prolif : Focal mesangial proliferation**

**Tubular changes: cloudy swelling**

**I.s inflam: interstitial inflammation**

**I.s cong: interstitial congestion**
